# The Climate of Puerto Rico

**Published:** 1900-05

**Authors:** Charles J. Proben

**Affiliations:** New York


ATLANTA

### Journal-Record of Medicine.

**Successor to Atlanta Medical and Surgical Journal, Established 1855, and Southern Medical Record, Established 1870.**

VOL. II. MAY, 1900. No. 2.

EDITORS { BERNARD WOLFF, M.D., DUNBAR ROY, M.D.

M. B. HUTCHINS, M.D., BUSINESS MANAGER.

PUBLISHED MONTHLY. OFFICE 305 AND 306 FITTEN BUILDING.

### *ORIGINAL COMMUNICATIONS.*

### THE CLIMATE OF PUERTO RICO.

BY CHARLES J. PROBEN, M.D.,

NEW YORK.

The inclement weather, as experienced throughout the Atlantic section of our States part of the winter, caused a great deal of suffering and unwonted hardships to man and beast. With a persistent attitude of the mercury in the barometer below the zero mark, the extreme cold with the prevailing northwest winds forcibly and unregardedly permeated the palatial mansion with its cheerful fireside, equally as well as the roughly constructed hut or hamlet.

Those improperly sheltered suffered severely from the intense cold, especially if duty compelled their presence out of doors; the better housed and clothed were not entirely exempt from their association with permeating Jack Frost. Contrasting these conditions with those existing at the same time in our recently acquired possessions, what a meteorological gulf looms up between the two.

Noble participants of the late war, who so valiantly exemplified the supremacy of the Anglo-Saxon race, were constrained, by reason of conquest, to inhabit territory, whose physical conditions were radically different from those to which they had for a lifetime become accustomed to.

A sudden change of residence to a tropical climate, frequently necessitated in the course of a few days, with arduous duties attached, would appear to induce the average constitution to rebel against any such sudden radical intrusion. This, however, did not primarily seem to be the experience of the majority, as there was a surprisingly well borne toleration of the hot climate.

Uncle Sam can now verily boast, that, in his large and mighty domain, he has all kinds of degrees of weather and climate on tap; amply sufficient to satisfy the tastes of the most fastidious and the most exacting of mankind. Including among these rather recently acquired territories in the far *North* and the far *South,* irrespective of season, he can offer the tourist any choice of climate he may desire. Abounding in nature's vast wonders and prolific of primitive creation; with sceneries of grandeur, our country really offers unusual inducements for travel and for those in quest of pleasure and sightseeing, who scarcely need henceforth cross the Atlantic in order to satisfy their desire for such.

Situated right in the center of nature's pleasure-ground, the excursionist need scarcely be attracted to European shores; a trip entailing innumerable inconveniences in the face of staring, sumptuous and excessive disbursements. When the powerful arm of our Republic has fully induced strife to be subservient to order and tranquillity, avenues for travel will readily be thrown open, affording the pleasure-seeker an opportunity for renewed interest to explore the tropical countries situated right at our very doors. The arousal of renewed interest for exploration of these possessions will presumably divert a part of the annual exodus of travel and somewhat curtail those exacting and abusive restrictions so freely levied upon the rich American tourist in Europe.

Even in spite of the martial decree imposed upon the traveling public to wend it way to European climes, it is to be hoped that in future the Atlantic and Pacific coasts will team with modern greyhounds ready to convey a willing public to our new colonial possessions. The recent territorial acquisitions in the far North have not awakened that interest which the tropical possessions undoubtedly will, owing to the reluctance of the white man to freely expose himself to a frigid temperature of probably 50 degrees below zero, merely for the sake of pleasure.

Abode in a moderately warm climate far better suits the requirements of the Caucasian, who seeks relaxation and diversion from his arduous business cares. The mildness of the climate of Puerto Rico as favorably impressed the first discoverers as the beauty and the grandeur of this delightful island.

Ponce de Leon describes the island as a veritable "Garden of Eden" in which he foresaw visions of the long-sought for "Fountain of Youth," and in consequence of which he selected it as his abode and built the substantial structure called the "Governor's Palace" or the "Casa Blanca" within the "Santa Catalina" fortifications at San Juan. From the advent of Spanish conquest to the present time, this "Pearl of the Antilles" has been reputed to be an exceedingly salubrious island. As the corrupt and greedy Dons were complete rulers, they kept constantly enriching themselves by following policies of avarice; neglectfully failing to provide any means for sanitary improvements. Even in spite of such neglect the island has retained its former reputation of salubriousness to man.

Suddenly an alarming amount of sickness appeared among the troops of the invading army of the United States, which immediately caused a cry of unhealthfulness of the island. True, certain kinds of fevers are to be expected in the tropics, but the percentage really ascribed to the climate was relatively small in Puerto Rico.

At first almost every sickness was attributed to climate, but abode was the only exciting etiological factor. Consideration should have been given to the sudden and radical transition which numerous recruits were subjected to, unaccustomed to camp life and especially to a sudden change of diet, unsuitable for the digestive organs of those whose functions have become impaired by the continuous heat.

In the tropics the system must necessarily undergo a process of acclimation, which physically produces a certain amount of deterioration, making it less capable of coping with disease. Following the customs and usages of tropical countries, the relative proportion of carbohydrate and proteid foodstuffs should have been approximately adjusted for our soldiers. To the uninitiated, the ingestion of the majority of tempting tropical fruits played havoc with the digestive organs, especially in conjunction with a failure to observe the ordinary laws of hygiene.

Segregation of a large number of men in a campaign always awakens a tendency to the development of much sickness from slight causes. If coincidently infectious diseases are introduced into a new country, the spread is apt to be alarming and its virulence notably enhanced. It will be seen that numerous factors contribute to the causes producing the larger percentage of sickness among our troops in Puerto Rico, as high as 35 per cent. at one time, which was primarily attributed to the climate, considered as insalubrious to man, an assumption exploded by four centuries' habitation, devoid of any such past reputation, in spite of wilful neglect to provide proper sanitary means, as modern custom and experience would dictate.

Regarding the physical characteristics of Puerto Rico, its topography suggests ample climatological advantages; an island favorably situated and lengthwise traversed by a chain of mountains, reaching an altitude of four thousand feet in its interior, with a gradual slope toward the coasts, offers as perfect a shed for drainage as a gable roof would.

Intersected by forty-seven large rivers and over three hundred smaller ones, issuing from the mountains, pure drinking-water is conveyed within the reach of all. This superabundance of pure water, materially increased by large fire forest destruction, was known to ancient mariners and to the inhabitants of adjacent islands, who were wont to come great distances for a supply. The part this abundance of water plays in the vegetation of the island is more than a matter of conjecture. Arability of the soil is attested by the constant verdure of the meadows and the fertile fields with the rapid growth of the large Graminaceæ amid the majestic palms which afford conspicuous instances of the power of "Old Sol," when the soil is properly supplied with copious moisture. The climate would be intensely hot, sultry and almost intolerable were it not for the presence of currents of cool sea air, and the lessened relative humidity which tend to mitigate the effects of the heat. These factors materially tend to inhibit the effects of the heat and make the climate more endurable, bringing about a mild, soothing, balmy and bearable atmosphere. Humidity is the terrible fiend in our climate during the summer months, and one who has repeatedly experienced the hot, muggy day of the metropolis knows how exasperating, prostrating and disagreeable they are to encounter. While along the New England coast and inland these days are comparatively frequent, they are but relatively infrequent in Puerto Rico.

The average person from the temperate zone, emigrating to this island, bears the climate remarkably well, and far less inconvenience results than is anticipated. Before the system becomes fully acclimated however, relaxing and enervating effects are apt to be encountered, though this is really less severe than one would expect. Coming from the sea the equatorial trade-winds constantly blow over the southern coast. Inland, mountain and valley winds exist, while along the northern coast of the island a sea breeze, induced by currents of the Gulf Stream, holds sway.

It is invariably the rule, that from sunrise to sunset, a constant, continuous and steady sea breeze sweeps over the island, which cools the atmosphere and makes the climate rather agreeable. Besides attributing much of this current to trade-winds, part is due to the suction produced by attenuated air rising from the heated earth's crust, which again becomes cooled after sunset. The water, which offers different physical conditions, retains its specific heat longer, and consequently, if more or less stagnant, feels comparatively warm to the bather.

During the day the breezes in the mountains are refreshing; the nights appear cool, heavy fogs settle in the valleys, to again become dispersed by the fervid sun's rays. Though the island is thickly populated, its relative freedom from dust and germ-life can be accounted for by the population being promiscuously scattered among nature's lavishly bestowed vegetation and by the constancy of the breezes.

Manufactories and similar industries, which so freely contaminate the atmosphere of our large cities, are conspicuous by their almost total absence. An atmosphere laden with oxygen and ozone is always plentiful where plant life is prolific.

Good weather is the rule, though one disadvantage of a residence appears to be the rainy season, which is ushered in about the middle of August and persists almost uninterruptedly for three months. This does not mean that rain is constant, but when it does come down it is apt to become a deluge. Daily showers usually occur, appearing just as suddenly as they may abruptly terminate, to be followed by bright sunshine. Rain, on the other hand, may fall incessantly for one, two or three days, accompanied by furious winds and deluge everything, inundating fields, creating swamps and lagoons and swelling rivers to an alarming extent. From a harmless inert fordable stream an impassable, rushing river may be created; the impediments thus offered to traffic along the miserable country roads can well be imagined.

The mean average fall for the months of August, September and October have respectively been estimated to be 6.65 inches; 5.30 inches and 7 inches. The natives can accurately foretell the rapid rising and swelling of the stream after a heavy rain, by perceiving small pieces of driftwood approaching in an accelerated current; thus warning inexperienced travelers to delay hazardous fording, which otherwise would be the cause of being caught in a dangerous and destructive current. The forcible mill-race current may tear away huge masses of earth, trees and bridges and act very destructively to anything in its path. Absence of bridges will frequently cause the travelers to become entrapped for a number of days before subsidence of the flood can be expected. Even with a rather strong and dangerous current the colored native will unceremoniously disrobe and attempt to drag horse and rider across at great risk to himsef, selecting some known path and soliciting a small remuneration for his gallant services. It is a rather novel sight, amusing and impressive, to see a nude native prepared to forewarn and ready to attempt to take rider across the swollen waters, though hazardous it may appear. The River Portuguez, adjoining the encampment of the U. S. engineers, rose, after a heavy rain in the mountains, by actual measurement 14 feet in forty-five minutes, inundating the camp site, which necessitated removal of tentage and troops until subsidence of the flood. This occurred very suddenly and with practically no forewarning.

It is related that a flood occurred in the same locality a few years ago, devastating and destroying much property and sacrificing over one hundred lives. With a heavy rainfall it is not to be wondered at that persons exposed will become quickly drenched to the skin, the apparent warmth of the air usually preventing chilling effects. Moisture during the day is less detrimental than at night, especially if the person is compelled to sleep on the bare ground with inadequate protection, which rapidly impairs his vigor and makes the system a ready prey for paludal poison. Assuming how detrimental such exposure would be to the pulmonary organs of the Anglo-Saxon in his own climate, we find practically that an immunity exists against such affections in the tropics.

Of all the aerial phenomena feared by the natives, none is more dreaded than the West India cyclone, which at times visits Puerto Rico, though this does not exactly lie in it natural path. The mariner well knows its devastating effects, for in its fury it respects neither life, limb, habitation nor vessel. The native seeks adequate protection by properly constructing his houses of stone and cement, allowing huge openings for windows, barricaded with strong doors on the inside. Glass windows would offer little resistance to the fury of these winds, and partly also for economic purposes, glass windows are unknown in Puerto Rico, even in the larger cities, which are better civilized. So often are readers misinformed about the meteorological conditions of tropical countries, that erroneous theories become inculcated in their minds, which are radically at variance with existing facts. Puerto Rico, so often depicted as a garden spot of creation, must necessarily have something attractive in its climate to exert a rather favorable impression. The ocean breezes mitigating the hot summer days make the climate endurable, unoppressive and bearable. It may be said without any exaggeration that the soldier in Puerto Rico did not suffer as much from the heat as he would have at one of the national camp sites. The nights are, as a rule, cool, soothing and sleep-producing, rather the opposite of a muggy night in New York city, which produces physical debility and banishes sleep. Allusion has already been made to the debilitating and enervating effects experienced by the majority passing through a process of acclimation. As noticed in all tropical countries the heat fosters an aptitude for indolence and a reluctance for the performance of any severe manual labor. The majority of our soldiers in fact had arduous duties to perform, in comparison to the loath and lazy actions of the native laborer, and yet they bore the heat remarkably well; in fact, so much so, that not a single case of insolation or heatstroke was recorded in the regiment to which the writer was attached. This will more than favorably compare with the records of regiments stationed at Tampa, Chickamauga or Peekskill.

During the heated term of the day the white man is not slow in learning to adopt the abstemious habits of the natives and takes a quiet siesta, which seems to be refreshing and a necessary adjunct to life in tropical countries. Following this dormant attitude, activity asserts itself, and after dinner hour the evenings are devoted to amusements, promenading and visiting the shops to make the necessary daily purchases. The restless activity of our American business man, the keen commercial instinct, and the greed for wealth do not dominate the civic ideas of the native born; rather the opposite seems to prevail. Contentment in the face of barely sufficient subsistence and lack of ostentation are the rule. The pinnacle of ambition may have been seriously curtailed by long-continued Spanish misrule and oppression, though undoubtedly climate plays an important part in shaping the lazy, inactive policy of their very existence. No matter how mild and equable this appears to be, we must attribute a material influence of climate upon the habits of natives. In Puerto Rico it is the exception to have such extreme diurnal variations of temperature and sudden fluctuations as we frequently experience North. Transitions being more gradual are less detrimental to health than sudden extreme changes, especially if we reflect that the mean monthly temperature in Puerto Rico rarely varies more than two degrees from month to month. Approximately corresponding to our summer months 95 degrees F., and to our winter months 75 degrees F., is the rule at Ponce, Puerto Rico. A more detailed record of temperatures and some relative facts appear in the chart at the end of this paper. These records were taken in the shade, underneath a white canvas field-tent, at an altitude of two hundred and seventy-five feet, two miles west of Ponce, P. R. Further facts regarding meteorological conditions, though desirable, could not be ascertained, owing to the absence of instruments to measure rainfall, humidity and atmospheric pressure. Between the sun and shade temperature an approximate difference of fifteen degrees Fahrenheit exists.

For the month of September, 1898, we find the following mean daily temperature, taken five times a day at 6, 9, 12, 3 and 7 o'clock, to average as follows: 72 degrees F., 86 degrees F., 89 degrees F., 85 degrees F., 79¾ degrees F. The minimum temperature was recorded on the mornings of the sixth and of the twentieth day respectively, and is 68 degrees F. The maximum temperature occurred at noon of the twentieth day, and was 96 degrees F. in the shade, and 113 degrees F. in the sun. Covering a period of four months, the hottest months of the year, this was the highest temperature recorded.

The mean monthly temperature for September is 82½ degrees F., for October 80 degrees F., and for November 78 degrees F.

Cayey, a mountain resort near the Spanish stronghold, forty kilometers from the coast, situated at an altitude of twenty-three hundred feet and considered one of the coolest spots of the island, records an average summer temperature of from 77 to 82 degrees F.; the maximum being 87.8 degrees F. The average winter temperature recorded here, the lowest on the island, during the years 1897 and 1898 was 59 to 64 degrees F.; the minimum said to have been the lowest temperature recorded was 48 degrees F.

The maximum temperature obtained at Cayey by the Spanish engineers corresponded exactly with that which we observed at Ponce, namely, 96 degrees F. It will be apparent that the maximum figures favorably compare with those observed in our Temperate Zone during the heated term; in fact, they appear almost incredible when comparison is invited regarding equability and humidity of climate. A relatively large absolute percentage of humidity is what makes our climate so depressing to mankind. Scanning over the chart it will be observed that the greatest rise of temperature occurs during the three consecutive hours following six o'clock in the morning. Fourteen degrees rise is the record for this time in contradistinction to five degrees rise until noon, a time of the day in which the greatest range of variation naturally would be expected. From noon until sunset, covering a period of six hours, a decline of 10 degrees is invariably the rule. Deducting ten degrees from the temperature observed at sunset will approximately give the minimum temperature of the night.

The record of San Juan, the capital, situated on the northern coast of Puerto Rico, shows an average mean monthly temperature for August, September and October respectively as follows: 81 degrees F., 80.5 degrees F. and 79.5 degrees F.; the nocturnal mean temperature is 68 to 69 degrees F. Our winter months correspond to a constant summer in Puerto Rico; a summer resplendent with verdure, with an average temperature of 60 to 70 degrees F. In this realm it is correctly borne out by experience that with such an equable moderate temperature, burdensome clothing and top coats are cumbersome objects. The opposite extreme of dress prevails, so much so, as to excite the wonder of the stranger when he espies the piccaninny and the creole child running about unconcernedly, devoid of any clothing. This custom is universal among children; the little ones appear to thrive fairly well, irrespective of the vicissitudes of weather and exposure, in the presence of dirt and squalor; circumstances which we would unhesitatingly pronounce adverse to growth and development of children.

In a changeable climate like ours exposure would indeed be hazardous to the frail structure of infancy, aside from the moral outcry such custom undoubtedly would provoke. In the warm equable atmosphere of Puerto Rico exposure to rain will have little immediate effect upon the health, and need not detain the traveler, who rapidly learns by experience the harmlessness of exposure to inclement weather. The condition of the primitive paths and poor country roads, barring the finely macadamized military highway, show that but little attention has been bestowed upon the subject of road construction. The military road, transversing the island, a masterpiece of engineering, is well kept and affords exceptionally fine views of tropical country from the mountains, a trip well worthy of the time spent and which has been lauded with the most unstinted praise. Ordinarily the roads of Puerto Rico are execrable, especially if we consider their condition after a heavy rainfall and the impediments they offer to travel; such as the native bullock carts, which is the average means of transportation for the planters. Much of the poverty and lowness of the people can be attributed to an inability to rapidly dispose of their products by the primitive methods of travel and transportation facilities along the execrable road-beds.

Chilling effects of rain upon the skin are not as a rule encountered in a warm, equable atmosphere, a reason why relatively few pulmonary and rheumatic affections exist among the natives. Frail individuals, invalids and those similarly affected would undoubtedly derive benefit from a sojourn in a warm, even climate, where consideration can be given to the selection of a desired altitude and adaptability of an outdoor life. For a frail constitution to recuperate, especially if brought about by excessively imposed social exactions, which the Anglo-Saxon is more or less compelled to accept during the winter's season of dissipation, hardihood and exposure, with a more primitive form of existence, would indeed be beneficial. Adoption of more natural methods of living, with alleviation of these enervating social influences, away from a hothouse existence of artificiality, would rapidly transform many a weak, helpless individual, on the verge of collapse, to hardy manhood. At the decline of the century, when excessive social pleasures exert a marked tendency to deterioration of the race, caused to a large degree by exaggerated and excessive artificiality in living, an outdoor life, away from the turmoil of the city, would rapidly transform physically devitalized manhood into men worthy of their peers. A trip on horseback along the military road from Ponce to San Juan abounds in ever-appearing, exhilarating and instructive views of nature's lavish deeds in the tropics. Engineers versed in road construction are urgently needed in order to bring about means to facilitate travel for the planter, who, desirous of disposing of his products, encounters numerous impediments to transportation; which, as a consequence, cause stagnation of business enterprise and inhibition to commerce. In a country so fertile as Puerto Rico and with so docile a race, these factors call for speedy relief. Inadequate facilities have caused retrogression of the overtaxed and oppressed native, who has been kept in constant subjection and in a state of misery.

Many complex commercial and sociological problems for the relief and improvement of the race confront our government; one of the first to stimulate the inhabitants to renewed activity is to construct proper roads and provide adequate means for transportation. An intention of good-will, supplemented by rapid deeds, will arouse feelings of brotherhood and esteem in the oppressed native, who is honorably seeking his civic as well as his political liberty.

In a short time much has already been accomplished by a wise, conservative and tactful governor-general. Open up the avenues of trade for the rich tropical products, which our nation is urgently in need of, and commercial benefits will rapidly and mutually accrue. Acquisition of territory at first for humanitarian purposes is rapidly relegated to annexation and retention with a unity of existence and the freedom of a home for all. Liberal exports of the rich products of Puerto Rico are destined to shape a future policy and to make the island an integral part of the Union, in which we in future shall feel a profound civic pride.

RECORD OF TEMPERATURE AT PONCE, P. R., DURING SEPTEMBER, OCTOBER AND PART OF NOVEMBER.

*Taken at the Field Hospital, 1st Reg. U. S. V. Engrs., Altitude of Camp 117′ Above Sea Level. Chas. J. Proben, Assistant Surgeon.*

| DATE. | A. M. | | | P. M. | | FOR MONTH OF SEPTEMBER. | |
|---|---|---|---|---|---|---|---|
| | Number of Degrees. | | | | | Remarks. | Daily Average. |
| Sept. 3 | 73° | 86° | 90° | 88° | 76° | Clear and dry. Afternoon winds, south southeast .... | 82.6° |
| 4 | 70 | 86 | 88 | 84 | 80 | Clear. Light afternoon showers .......... | 81.6 |
| 5 | 73 | 86 | 90 | 87 | 78 | Clear and dry .......... | 82.8 |
| 6 | 73 | 88 | 90 | 88 | 76 | Clear and dry. 12 A. M. 104° in the sun .......... | 80 |
| 7 | 68 | 90 | 86 | 87 | 78 | Clear and dry .......... | 81.8 |
| 8 | 71 | 88 | 85 | 86 | 84 | Clear and dry. Afternoon showers .......... | 82.8 |
| 9 | 75 | 84 | 90 | 88 | 85 | Night showers .......... | 84.4 |
| 10 | 75 | 89 | 90 | 88 | 86 | .......... | 85.6 |
| 11 | 72 | 92 | 93 | 90 | 86 | .......... | 86.8 |
| 12 | 76 | 88 | 90 | 88 | 86 | .......... | 85.6 |
| 13 | 73 | 86 | 93 | 84 | 86 | .......... | 84.4 |
| 14 | 75 | 90 | 92 | 87 | 82 | .......... | 85.2 |
| 15 | 73 | 92 | 91 | 90 | 84 | .......... | 86.2 |
| 16 | 74 | 79 | 86 | 78 | 76 | .......... | 78.6 |
| 17 | 76 | 84 | 88 | 88 | 80 | .......... | 83 2 |
| 18 | 76 | 87 | 91 | 84 | 78 | .......... | 83.2 |
| 19 | 72 | 82 | 88 | 81 | 76 | .......... | 79.8 |
| 20 | 68 | 88 | 96 | 80 | 78 | 12 A. M. in sun 113°. Severe thunder storm—P. M ...... | 82 |
| 21 | 70 | 91 | 92 | 82 | 78 | .......... | 82.6 |
| 22 | 71 | 78 | 76 | 73 | 72 | Heavy showers all day .......... | 73.8 |
| 23 | 70 | 74 | 75 | 72 | 70 | .......... | 72.8 |
| 24 | 74 | 82 | 86 | 84 | 79 | .......... | 81 |
| 25 | 72 | 86 | 92 | 88 | 80 | .......... | 83.6 |
| 26 | 68 | 88 | 92 | 86 | 80 | .......... | 82.8 |
| 27 | 70 | 89 | 93 | 90 | 84 | .......... | 85.2 |
| 28 | 70 | 88 | 94 | .. | .. | .......... | 84 |
| 29 | 70 | 86 | 91 | 94 | .. | Monthly average—6 A. M. 9 A. M. 12 3 P. M. 7 P. M. | 84.2 |
| 30 | 72 | 84 | 92 | 80 | 76 | 72.536. 86.143. 89.286. 84.9. 79.777. | 80.8 |

Total Monthly Average, 82.528.

### RECORD OF TEMPERATURE—CONTINUED.

| DATE. | | A. M. | | | P. M. | | FOR MONTH OF OCTOBER. | |
|---|---|---|---|---|---|---|---|---|
| Number of Degrees. | | | | | | | Remarks. | Daily Average. |
| Oct. | 1 | 74° | 86° | 88° | 86° | 74° | ................................................ | 81.6° |
| | 2 | 70 | 88 | 88 | 82 | 76 | Severe rain storm.................................... | 80.8 |
| | 3 | 68 | 86 | 92 | 80 | 76 | Rain storm............................................ | 80.4 |
| | 4 | 70 | 76 | 78 | 78 | 77 | ................................................ | 75.8 |
| | 5 | 72 | 83 | 82 | 78 | 74 | ................................................ | 77.8 |
| | 6 | 68 | 78 | 80 | 80 | 96 | ................................................ | 76.4 |
| | 7 | 76 | 76 | 80 | 82 | 78 | ................................................ | 78.4 |
| | 8 | 74 | 32 | 85 | 80 | 76 | ................................................ | 79.4 |
| | 9 | 74 | 85 | 87 | 84 | 78 | ................................................ | 81.8 |
| | 10 | 70 | 84 | 88 | 78 | 76 | ................................................ | 79.2 |
| | 11 | 72 | 86 | 88 | 86 | 78 | ................................................ | 82 |
| | 12 | 72 | 86 | 86 | 84 | 78 | ................................................ | 81.2 |
| | 13 | 68 | 84 | 94 | 86 | 80 | ................................................ | 80 |
| | 14 | 74 | 86 | 86 | 82 | 78 | ................................................ | 81.2 |
| | 15 | 74 | 86 | 87 | 85 | 80 | ................................................ | 82.4 |
| | 16 | 72 | 87 | 88 | 87 | 70 | ................................................ | 80.8 |
| | 17 | 68 | 87 | 88 | 86 | 78 | ................................................ | 81.4 |
| | 18 | 70 | 84 | 90 | 86 | 82 | ................................................ | 80.4 |
| | 19 | 70 | 82 | 88 | 80 | 72 | ................................................ | 78.4 |
| | 20 | 70 | 85 | 80 | 78 | 72 | ................................................ | 78.2 |
| | 21 | 68 | 80 | 86 | 82 | 80 | ................................................ | 79.2 |
| | 22 | 72 | 85 | 88 | 88 | 84 | ................................................ | 79.4 |
| | 23 | 68 | 86 | 88 | 86 | 80 | ................................................ | 81.6 |
| | 24 | 70 | 87 | 89 | 84 | 78 | ................................................ | 81.6 |
| | 25 | 72 | 86 | 86 | 80 | 72 | ................................................ | 79.2 |
| | 26 | 67 | 83 | 88 | 82 | 74 | ................................................ | 78.8 |
| | 27 | 67 | 85 | 87 | 80 | 72 | ................................................ | 78.2 |
| | 28 | 66 | 84 | 85 | 82 | 74 | Monthly average—6 A. M. 9 A. M. 12 3 P. M. 7 P. M. | 72.2 |
| | 29 | 70 | 85 | 88 | 87 | 76 | 70.551. 84.104. 86.69. 82.828. 76.517. | 81.2 |

Total Monthly Average, 80.138.

FOR MONTH OF NOVEMBER.

| | | | | | | | | |
|---|---|---|---|---|---|---|---|---|
| Nov. | 1 | 72° | 84° | 88° | 74° | 72° | .............................................................. | 78° |
| | 2 | 70 | 82 | 88 | 76 | 74 | .............................................................. | 78 |
| | 3 | 72 | 84 | 88 | 80 | 74 | .............................................................. | 79.6 |
| | 4 | 72 | 83 | 86 | 84 | 76 | .............................................................. | 80.2 |
| | 5 | 72 | 84 | 8[illegible] | 82 | 76 | .............................................................. | 80 |
| | 6 | 68 | 82 | 88 | 80 | 72 | .............................................................. | 78 |
| | 7 | 68 | .. | .. | .. | .. | Temperature and barometer pressure at Cayey, Puerto Rico. Elevation 1,300 ft. above sea level. | .... |

From observations of Spanish Military Engineers. Records obtained by

DR. CHARLES J. PROBEN, 1st U. S. V. Eng'rs.

---

| | | | | |
|---|---|---|---|---|
| Average summer temperature...................... | 25° to 28°. | Cent. | = 77° to 82.4° | Fahr. |
| Maximum obtained temperature..................... | 31 | " | = 87.8 | " |
| Average winter temperature........................ | 15 | " | = 59 " 64 | " |
| Minimum obtained temperature..................... | 8 | " | = 48 4 " | " |

Average of barometric observations, 59.30°. Corresponding to 59.80° at sea level.

